# MicroRNA profiles in aqueous humor between pseudoexfoliation glaucoma and normal tension glaucoma patients in a Korean population

**DOI:** 10.1038/s41598-022-09572-4

**Published:** 2022-04-13

**Authors:** Hyun-kyung Cho, Hyemin Seong, Changwon Kee, Dae Hyun Song, Seong Jae Kim, Seong Wook Seo, Sang Soo Kang

**Affiliations:** 1 Department of Ophthalmology, Gyeongsang National University Changwon Hospital, Gyeongsang National University, School of Medicine, 11 Samjeongja-ro, Seongsan-guGyeongsangnam-do, Changwon, 51472 Republic of Korea; 2 Institute of Health Sciences, School of Medicine, Gyeongsang National University, Jinju, Republic of Korea; 3 Department of Pharmacology and Convergence Medical Science, School of Medicine, Gyeongsang National University, Jinju, Republic of Korea; 4 Department of Ophthalmology, School of Medicine, Gyeongsang National University Hospital, Gyeongsang National University, Jinju, Republic of Korea; 5 Department of Ophthalmology, Samsung Medical Center, Sungkyunkwan University School of Medicine, Seoul, Republic of Korea; 6 Department of Pathology, Gyeongsang National University Changwon Hospital, Changwon, Republic of Korea; 7 Department of Anatomy and Convergence Medical Science, Gyeongsang National University, Jinju, Republic of Korea

**Keywords:** Genetics, Clinical genetics, Clinical epigenetics, Optic nerve diseases

## Abstract

We aimed to obtain microRNA (miRNA) profiles of patients with pseudoexfoliation (PEX) glaucoma or normal-tension glaucoma (NTG) compared to normal controls using individual aqueous humor (AH) samples and investigate the role of miRNAs in the pathogenesis of PEX glaucoma compared to NTG in Korean. AH (80-120 µl) was collected before cataract surgery or trabeculectomy from 26 Korean subjects (eleven with PEX glaucoma, age-matched eight NTG, and seven controls). RNA sequencing was conducted for RNA samples extracted from 26 AH samples. Bioinformatics analysis was performed for targets and related pathways. A total of 334 and 291 discrete miRNAs were detected in AH samples of PEX glaucoma and NTG patients, respectively. Two significantly upregulated miRNAs (hsa-miR-30d-5p and hsa-miR-320a) and ten significantly downregulated miRNAs (hsa-miR-3156-5p, hsa-miR-4458, hsa-miR-6717-5p, hsa-miR-6728-5p, hsa-miR-6834-5p, hsa-miR-6864-5p, hsa-miR-6879-5p, hsa-miR-877-3p, hsa-miR-548e-3p, and hsa-miR-6777-5p) in PEX glaucoma patients compared to control (fold-change > 2, *p* < 0.05) were found. In NTG patients, ten significantly upregulated and two downregulated miRNAs compared to control were found. Only hsa-miR-6777-5p was commonly downregulated in both PEX glaucoma and NTG patients. Related pathways were proteoglycans in cancer, glioma, and TGF-beta signaling pathway in PEX glaucoma. These differentially expressed miRNAs between PEX glaucoma and NTG samples suggest the possible role of miRNA in the pathogenesis of glaucoma, further implying that pathogenic mechanisms may differ between different types of glaucoma.

## Introduction

Glaucoma, the second most common cause of visual impairment in the world, can lead to blindness^1,2^. The current estimated global population of primary open angle glaucoma (POAG) is 68.56 million (95% CI 59.99–79.98)^1^. Besides, Asians account for half (53.81%) of POAG patients^1^. Glaucoma is a neurodegenerative disorder. Its pathological feature is death of retinal ganglion cell (RGC)^3^. Pseudoexfoliation (PEX) syndrome is the most common identifiable cause of secondary glaucoma, named PEX glaucoma^4^. PEX syndrome is a disorder related with age and of the extracellular matrix (ECM) with the progressive accumulation and production of characteristic small, white deposits of a fibrillar material in many intra- and extra-ocular tissues^4^. Microscopic findings of this accumulation show granular amyloid-like protein fibers (glycoprotein). PEX glaucoma usually demonstrates higher intraocular pressure (IOP). It is characteristically more aggressive and less responsive to hypotensive anti-glaucoma medications than POAG^4^. The etiology of PEX syndrome is not yet discovered much, although there is speculation that it might have a genetic basis^5–7^. Genetic background differences among individuals could explain the different worldwide prevalence of PEX syndrome and glaucoma^5–7^ to some extent.

The association between lysyl oxidase-like 1 (LOXL1) gene and PEX syndrome or glaucoma has been widely investigated. Many single nucleotide polymorphisms (SNPs) in different ethnicities have also been researched^5,7,8^. However, there is no single disease-associated variant existent in all ethnic populations. Moreover, the disease-risk allele disagrees in diverse populations, particularly for LOXL1 risk alleles. Some alleles are flipped in Asians compared to Caucasians, while others are flipped in African descents compared to Caucasians^9,10^. The frequency of LOXL1 disease-associated SNPs also does not have tendency with the prevalence of the disease either^11^. Therefore, it is difficult to explain the pathophysiology of PEX syndrome and glaucoma with LOXL1 gene alone. Other than the role of one particular gene, epigenetic regulation of diverse gene expression is also thought to play a role in the pathogenesis of PEX syndrome or glaucoma^12^.

MicroRNAs (miRNAs) are noncoding small (~ 18 to 22 nt) oligoribonucleotides that can regulate post-transcriptional modulation of gene expression via the recognition of particular sequences in target mRNAs^13^. These miRNAs act principally to reduce the expression of target gene, which can eventually act as an epigenetic factor^14^. In opposition to mRNAs, miRNAs have demonstrated remarkable stability within biofluids^15^. Aqueous humor (AH) has been proposed to have potential molecular biomarkers with substantial pathophysiologic relevance^16^. Former studies have detected microRNAs in the AH of normal subjects and patients with POAG^17–20^. The expression of miRNAs in the AH of patients with exfoliation glaucoma or POAG has been reported^21^. However, that study included mainly Caucasians and some African descents. Among open angle glaucoma, the prevalence of normal-tension glaucoma (NTG) is higher in Asians than in other ethnicities, which is a distinguishing feature^22^. We have previously reported miRNA profiles from AH of NTG compared to controls in Asians (Koreans)^23^. However, no previous study has inspected miRNA expression in the AH of PEX glaucoma and NTG patients compared to normal controls in a single ethnic group of Asians (Koreans).

The difference in the type of glaucoma among ethnicities implies that genetic factors are important in the development of glaucoma^24–26^. Furthermore, glaucoma family history is broadly acknowledged as one of risk factors for glaucoma^24–26^. Therefore, genetic background is regarded to be involved in the development of glaucoma and its pathogenesis^24–26^.

Preliminary previous studies pooled AH samples to acquire enough volume for analysis. Nevertheless, in the present study, we used RNA sequencing to analyze microRNA expression in individual AH samples. In the current study, we investigated the expression of miRNAs in the AH between PEX glaucoma and NTG patients compared to normal controls in a single ethnic group of Koreans. We intended to see differentially expressed miRNAs that might suggest a clue to the pathogenic mechanism of different types of glaucoma between PEX glaucoma and NTG, which is especially prevalent in Asians. These differentially expressed miRNAs might also provide a clue to the pathogenic mechanism difference between secondary glaucoma and primary glaucoma.

## Results

### Baseline characteristics and demographics of subjects

After quality check test, eleven PEX patients and age-matched eight NTG patients with seven control subjects were included in the final RNA sequencing. Demographics of included subjects are demonstrated in Table 1. The mean age was 69.0 ± 23.3 years for PEX glaucoma subjects (n = 11), 67.0 ± 25.6 years for NTG subjects (n = 8), and 63.9 ± 9.9 years for control subjects (n = 7). The mean baseline IOP was 25.4 ± 9.2 mmHg for PEX glaucoma subjects, 14.4 ± 1.9 mmHg for NTG subjects, and 16.1 ± 2.0 mmHg for control subjects. Baseline mean deviation (MD) was − 5.9 ± 4.8 dB in the NTG group and − 18.6 ± 10.5 dB in the PEX glaucoma group. PEX glaucoma patients were using multiple topical anti-glaucoma medications before trabeculectomy. All NTG patients were using just one topical medication, including fixed combination eye drops. These included subjects had no ocular comorbidities other than simple cataracts.

**Table 1:** Baseline characteristics and demographics of subjects.

Subject Number	Disease STATUS	Age, yr	Sex	Eye laterality	Baseline IOP, mm Hg	Topical medication	Type of surgery
1	PEX G	69	Female	Right	31	Dorzolamide/timolol	Trab
2	PEX G	65	Male	Left	17	Dorzolamide/timolol, brimonidine	Trab
3	PEX G	67	Male	Right	17	Dorzolamide/timolol, brimonidine, bimatoprost	Trab
4	PEX G	55	Male	Left	35	Brinzolamide/timolol, brimonidine, Tafluprost	Trab
5	PEX G	64	Male	Left	37	Dorzolamide/timolol, brimonidine, travoprost	Trab
6	PEX G	83	Male	Right	20	Dorzolamide/timolol, brimonidine, latanoprost	Trab
7	PEX G	57	Male	Right	36	Brinzolamide , timolol	Trab
8	PEX G	89	Female	Right	25	Dorzolamide/timolol, brimonidine, bimatoprost	Trab
9	PEX G	63	Male	Right	18	Dorzolamide/timolol	Phaco + PCL
10	PEX G	78	Male	Left	11	Dorzolamide/timolol	Phaco + PCL
11	PEX G	86	Female	Right	32	Dorzolamide/timolol, brimonidine, latanoprost	Trab
12	NTG	56	Female	Left	13	Latanoprost	Phaco + PCL
13	NTG	57	Male	Left	13	Latanoprost	Phaco + PCL
14	NTG	55	Male	Left	17	Dorzolamide/timolol	Phaco + PCL
15	NTG	77	Female	Right	14	Dorzolamide/timolol	Phaco + PCL
16	NTG	72	Female	Right	15	Latanoprost	Phaco + PCL
17	NTG	76	Female	Right	17	Tafluprost	Phaco + PCL
18	NTG	76	Female	Right	14	Latanoprost	Phaco + PCL
19	NTG	74	Female	Left	12	Dorzolamide/timolol	Phaco + PCL
20	Control	75	Female	Left	14	None	Phaco + PCL
21	Control	57	Female	Right	14	None	Phaco + PCL
22	Control	76	Female	Right	15	None	Phaco + PCL
23	Control	54	Male	Left	18	None	Phaco + PCL
24	Control	53	Male	Right	16	None	Phaco + PCL
25	Control	71	Female	Right	17	None	Phaco + PCL
26	Control	61	Male	Right	19	None	Phaco + PCL

IOP; Intraocular pressure, NTG; Normal tension glaucoma, PEX; Pseudoexfoliation, G; glaucoma, Phaco + PCL; Phacoemulsification and posterior intraocularlens insertion, Trab; trabeculectomy.

### Differential miRNA expression in aqueous humor from patients with pseudoexfoliation glaucoma and normal tension glaucoma using RNA sequencing

**Figure 1:**
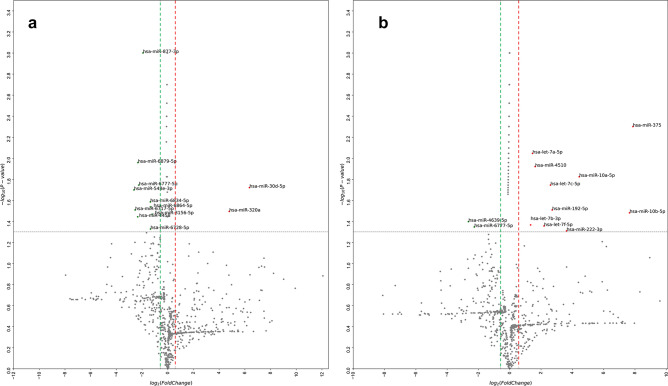
Volcano plot of miRNA expression in aqueous humor from subjects with pseudoexfoliation glaucoma and NTG compared to unaffected controls. (**a**) A total of 334 mature miRNAs were identified in the AH of PEX patients. Up-regulated miRNAs are shown to the right region of the plot (red) and down-regulated miRNAs are shown to the left region of the plot (green). Of these, two miRNAs, hsa-miR-30d-5p and hsa-miR-320a, were significantly up-regulated (fold-change > 2, *p* < 0.05) and ten miRNAs (hsa-miR-3156-5p, hsa-miR-4458, hsa-miR-6717-5p, hsa-miR-6728-5p, hsa-miR-6834-5p, hsa-miR-6864-5p, hsa-miR-6879-5p, hsa-miR-877-3p, hsa-miR-548e-3p, and hsa-miR-6777-5p) were significantly down-regulated (fold-change < − 2, *p* < 0.05) compared to controls. (**b**) In NTG patients, a total of 291 mature miRNAs were identified in the AH. Among these, ten miRNAs (hsa-let-7a-5p, hsa-let-7c-5p, hsa-let-7f.-5p, hsa-miR-192-5p, hsa-miR-10a-5p, hsa-miR-10b-5p, hsa-miR-375, hsa-miR-4510, hsa-let-7b-3p, and hsa-miR-222-3p) were significantly up-regulated (fold-change > 2, *p* < 0.05) and two miRNAs (hsa-miR-4639-5p and hsa-miR-6777-5p) were significantly down-regulated (fold-change < − 2, *p* < 0.05) compared to controls. Plots were prepared with ExDEGA v1.2.1.0 software. NTG: normal tension glaucoma, AH: aqueous humor, PEX: pseudoexfoliation.

Based on data from miRWalk 2.0, miRNA targets were analyzed. A total of 2,588 miRNAs were tested by RNA sequencing. A total of 334 mature miRNAs were identified in the AH of PEX patients (Fig. 1a). Up-regulated miRNAs are shown to the right region of the plot (red) while down-regulated miRNAs are shown to the left region of the plot (green). Of these, two miRNAs, hsa-miR-30d-5p and hsa-miR-320a, were significantly up-regulated compared to controls (fold-change > 2 or < − 2, *p* < 0.05). Ten miRNAs were significantly down-regulated compared to the controls (fold-change > 2 or < − 2, *p* < 0.05), which were hsa-miR-3156-5p, hsa-miR-4458, hsa-miR-6717-5p, hsa-miR-6728-5p, hsa-miR-6834-5p, hsa-miR-6864-5p, hsa-miR-6879-5p, hsa-miR-877-3p, hsa-miR-548e-3p, and hsa-miR-6777-5p.

**Figure 2:**
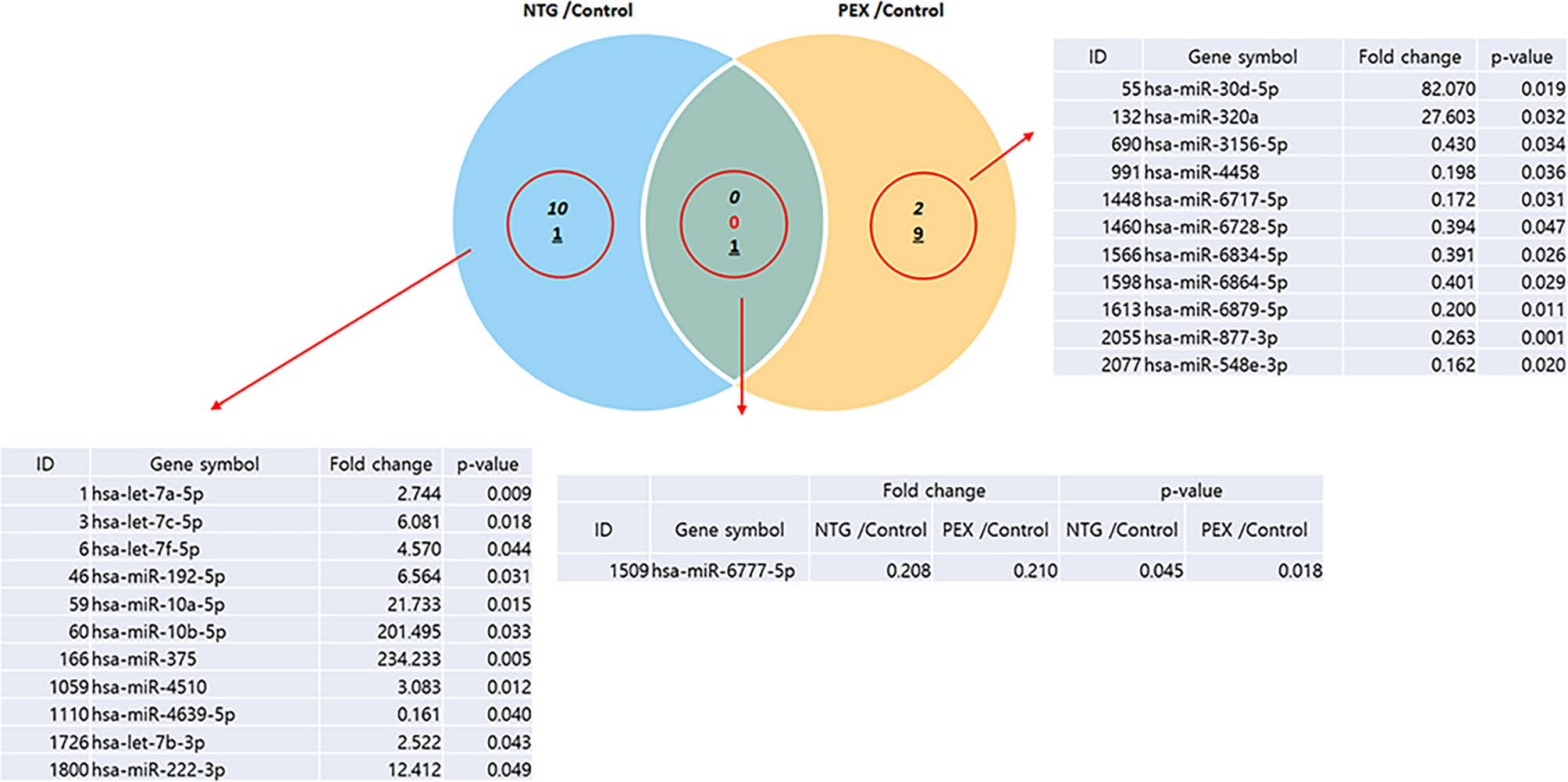
Venn diagram illustrating miRNAs significantly changed in PEX glaucoma and NTG samples versus control. Venn diagram shows the distribution of miRNAs between PEX glaucoma and NTG groups. The numbers in the figure represents the number of significant miRNAs detected within and between each group. Only hsa-miR-6777-5p, which was significantly down-regulated, was in common between PEX glaucoma and NTG groups. Other significantly differentially expressed miRNAs were not overlapped between the two groups of PEX glaucoma and NTG. PEX: pseudoexfoliation; NTG: normal tension glaucoma.

A total of 291 mature miRNAs were identified in the AH of NTG patients (Fig. 1b). Among these, ten miRNAs (hsa-let-7a-5p, hsa-let-7c-5p, hsa-let-7f.-5p, hsa-miR-192-5p, hsa-miR-10a-5p, hsa-miR-10b-5p, hsa-miR-375, hsa-miR-4510, hsa-let-7b-3p, and hsa-miR-222-3p) were significantly up-regulated and two miRNAs (hsa-miR-4639-5p and hsa-miR-6777-5p) were significantly down-regulated compared to those in controls (fold-change > 2 or < − 2, *p* < 0.05). (fold-change > 2 or < − 2, *p* < 0.05). Venn diagram (Fig. 2) showed the distribution of miRNAs between PEX glaucoma and NTG groups. Values in the figure represent the number of unique miRNAs identified within and between each group. Only hsa-miR-6777-5p, which was significantly down-regulated, was commonly found in both PEX glaucoma and NTG groups compared to controls. Other significantly differentially expressed miRNAs were not overlapped between the two groups of PEX glaucoma and NTG.

**Figure 3:**
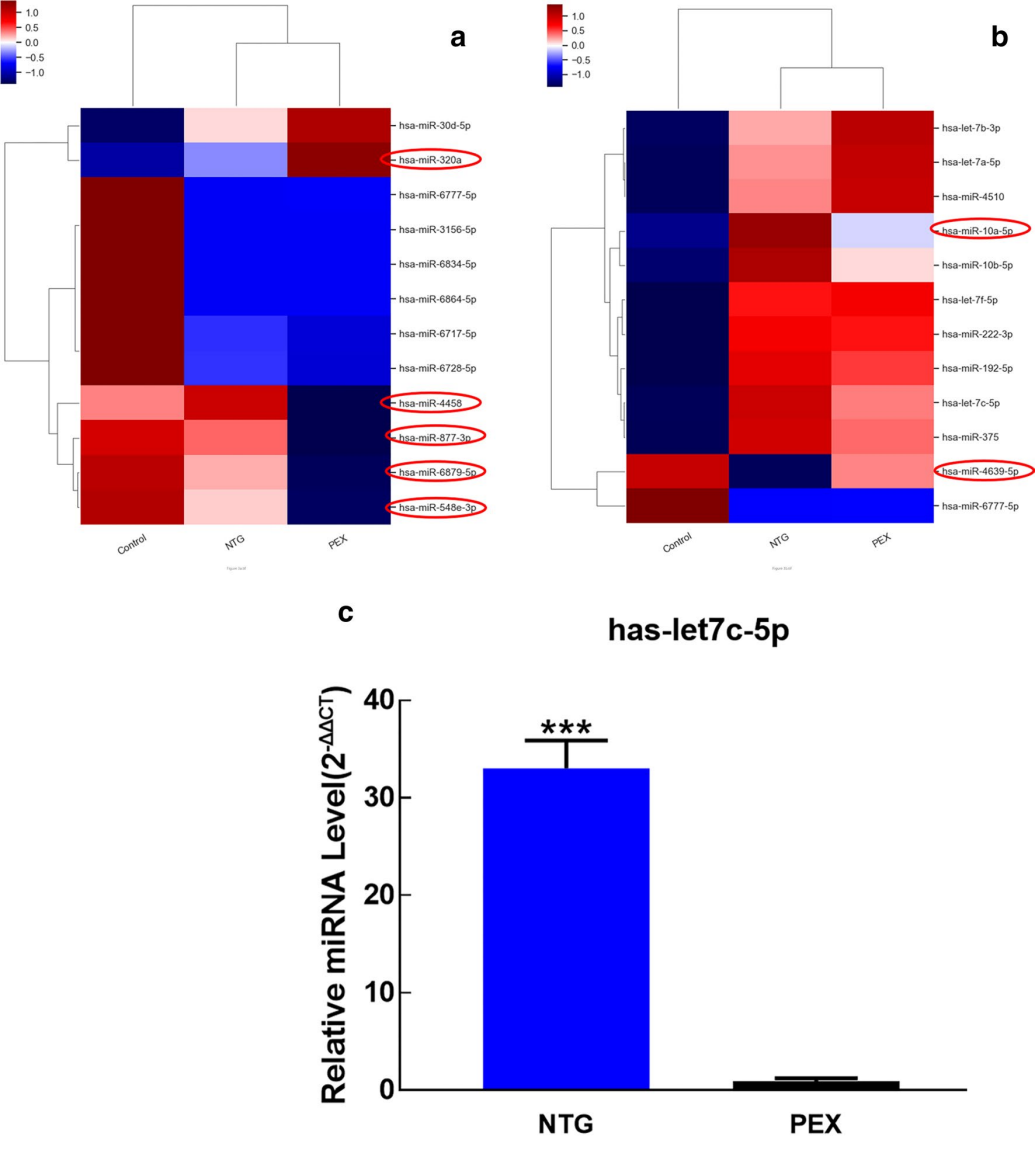
Heatmap diagram illustrating significantly differentially expressed miRNAs in PEX glaucoma and NTG. (**a**) A heatmap diagram demonstrates 12 significantly differentially expressed miRNAs in AH of PEX glaucoma patients (red, high relative expression; blue, low relative expression) compared to controls. They are shown in comparison with NTG patients. Among these 12 miRNAs, five miRNAs (hsa-miR-320a, hsa-miR-4458, hsa-miR-877-3p, hsa-miR-6879-5p, and hsa-miR-548e-3p) demonstrated opposite up/down regulated expression between PEX glaucoma and NTG. Although hsa-miR-320a was significantly up-regulated in PEX glaucoma, it was significantly down-regulated in NTG compared to controls. In cases of hsa-miR-4458, hsa-miR-877-3p, hsa-miR-6879-5p, and hsa-miR-548e-3p, significant expression of regulation showed opposite pattern between PEX glaucoma and NTG (i.e., they were significantly down-regulated in PEX glaucoma, but significantly up-regulated in NTG compared to controls). (**b**) A heatmap showing those 12 significantly differentially expressed miRNAs in AH of NTG patients compared to controls. They are shown in comparison with PEX glaucoma patients. Among these 12 miRNAs, two miRNAs demonstrated opposite up/down regulated expression between NTG and PEX glaucoma. Although hsa-miR-10a-5p was significantly up-regulated in NTG, it was significantly down-regulated in PEX glaucoma compared to controls. In case of hsa-miR-4639-5p, it was significantly down-regulated in NTG, but significantly up-regulated in PEX glaucoma compared to controls. (**c**) Bar graphs showing validation qPCR results between PEX glaucoma and NTG compared to the control. To validate the results of RNA sequencing from the AH of PEX glaucoma and NTG patients, let-7c-5p was used for quantitative PCR (n = 11). Similar results were obtained. The expression of let-7c-5p was increased significantly in the AH of NTG patients (33.03 ± 2.84-fold, *p* < 0.001) compared to the control (1.00). However, the expression of let-7c-5p in the AH of the PEX glaucoma patients (0.90 ± 0.30-fold, *p* = 0.67) showed no significant difference compared to the control (1.00). Data are presented as mean ± S.E.M. They were analyzed using unpaired Student’s t-test (n = 11). ***, *p* < 0.001 vs. control. PEX: pseudoexfoliation; NTG: normal tension glaucoma; AH: aqueous humor; PCR: quantitative polymerase chain reaction; S.E.M: standard error of the mean.

A heatmap diagram was prepared to demonstrate those 12 significantly differentially expressed miRNAs in AH of PEX glaucoma patients (red, high relative expression; blue, low relative expression) compared to control and NTG patients (Fig. 3a). Among these 12 miRNAs, five miRNAs (hsa-miR-320a, hsa-miR-4458, hsa-miR-877-3p, hsa-miR-6879-5p, and hsa-miR-548e-3p) demonstrated opposite up/down regulated expression between PEX glaucoma and NTG. Figure 3b shows those 12 significantly differentially expressed miRNAs in AH of NTG patients compared to control and PEX glaucoma patients. Among these 12 miRNAs, two miRNAs (hsa-miR-10a-5p and hsa-miR-4639-5p) demonstrated opposite up/down regulated expression between NTG and PEX glaucoma.

### microRNA validation and biological interpretation of differentially expressed miRNAs

To verify results of RNA sequencing from the AH of PEX glaucoma and NTG patients, let-7c-5p was analyzed using quantitative PCR (qPCR) (n = 11). Similar results were obtained (Table 2). The expression of let-7c-5p was increased significantly in the AH of NTG patients compared to that in the control (33.03 ± 2.84-fold, *p* < 0.001). However, the expression of let-7c-5p in the AH of the PEX glaucoma patients showed no significant difference compared to that in the control (0.90 ± 0.30-fold, *p* = 0.67) (Fig. 3c).

**Table 2:** Differentially expressed miRNA in the Aqueous Humor of Pseudoexfoliation Glaucoma and Normal Tension Glaucoma Patients.

miRNA	Assay ID	Accession Number	Fold change (Log2)	*P* value	Expression change
			PEX G /Control	PEX G /Control	
hsa-miR-30d-5p	55	MIMAT0000245	82.070	0.019	Up
hsa-miR-320a	132	MI0000542	27.603	0.032	Up
hsa-miR-3156-5p	690	MIMAT0015030	0.430	0.034	Down
hsa-miR-4458	991	MI0016804	0.198	0.036	Down
hsa-miR-6717-5p	1448	MIMAT0025846	0.172	0.031	Down
hsa-miR-6728-5p	1460	MIMAT0027357	0.394	0.047	Down
hsa-miR-6777-5p	1509	MIMAT0027454	0.210	0.018	Down
hsa-miR-6834-5p	1566	MIMAT0027568	0.391	0.026	Down
hsa-miR-6864-5p	1598	MIMAT0027628	0.401	0.029	Down
hsa-miR-6879-5p	1613	MIMAT0027658	0.200	0.011	Down
hsa-miR-877-3p	2055	MIMAT0004950	0.263	0.001	Down
hsa-miR-548e-3p	2077	MIMAT0005874	0.162	0.020	Down

miRNA	Assay ID	Accession Number	Fold change (Log2)	*P* value	Expression change
			NTG /Control	NTG /Control	
hsa-let-7a-5p	1	MIMAT0000062	2.744	0.009	Up
hsa-let-7c-5p	3	MIMAT0000064	6.081	0.018	Up
hsa-let-7f.-5p	6	MIMAT0000067	4.570	0.044	Up
hsa-miR-192-5p	46	MIMAT0000222	6.564	0.031	Up
hsa-miR-10a-5p	59	MIMAT0000253	21.733	0.015	Up
hsa-miR-10b-5p	60	MIMAT0000254	201.495	0.033	Up
hsa-miR-375	166	MI0000783	234.233	0.005	Up
hsa-miR-4510	1059	MI0016876	3.083	0.012	Up
hsa-miR-4639-5p	1110	MIMAT0019697	0.161	0.040	Down
hsa-miR-6777-5p	1509	MIMAT0027454	0.208	0.045	Down
hsa-let-7b-3p	1726	MIMAT0004482	2.522	0.043	Up
hsa-miR-222-3p	1800	MIMAT0000279	12.412	0.049	Up

PEX; Pseudoexfoliation, NTG: normal-tension glaucoma.

**Figure 4:**
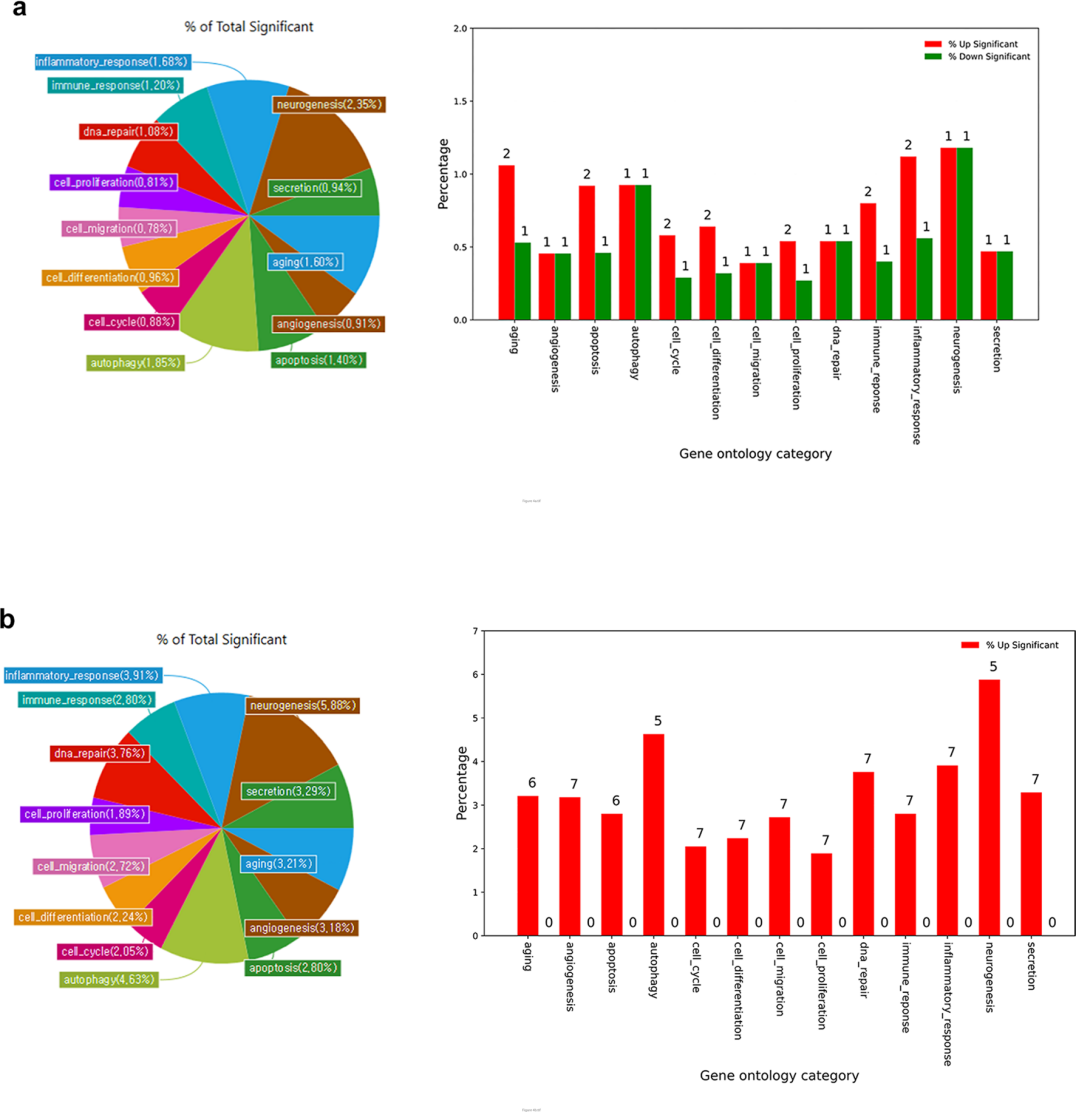
Percentage and number of microRNAs with significantly changed expression among gene ontology category-related microRNAs. Gene ontology categories of microRNAs with relatively large expression changes were identified in (**a**) PEX glaucoma and (**b**) NTG. Up-regulated miRNAs are shown as red graph and down-regulated miRNAs are shown as green graph. The percentage refers to the proportion of microRNA modified in AH of PEX glaucoma and NTG patients compared to controls in total miRNAs identified by researches in each GO category. (**a**) In PEX glaucoma, cell death-related categories including autophagy (1.85%) and apoptosis (1.40%) occupied the greatest proportion (3.25%). Categories related to neurogenesis (2.35%), inflammatory response (1.68%), and aging (1.60%) also presented significant proportions. In the process of randomly selected GO category analysis, only hsa-miR-30d-5p, hsa-miR-320a, and hsa-miR-877-3p were included among significantly differentially expressed miRNAs in PEX glaucoma. Many significantly differentially expressed miRNAs were not included in GO category analysis, indicating that these miRNAs were not previously reported in the field of miRNA. (**b**) In NTG, cell death-related categories including autophagy (4.63%) and apoptosis (2.80%) also occupied the greatest proportion (7.43%). Categories related to neurogenesis (5.88%), inflammatory response (3.91%), and aging (3.21%) also presented significant proportions. PEX: pseudoexfoliation; NTG: normal tension glaucoma.

To explore effects of these significantly differentially expressed miRNAs, gene ontology (GO) analysis was performed. Major GO categories of 15 were randomly chosen among many GO pathways. The percentage of total significant number of genes with differences in expression among each GO-related gene is presented in Fig. 4. The percentage refers to the proportion of microRNA altered in AH of NTG patients compared to that in the control in total miRNAs identified by research in each GO category. In PEX glaucoma, cell death-related categories, including autophagy (1.85%) and apoptosis (1.40%) occupied the greatest proportion (3.25%) (Fig. 4a). Categories related to neurogenesis (2.35%), inflammatory response (1.68%), and aging (1.60%) also presented significant proportions. Categories associated with cellular function, such as differentiation, migration, and proliferation that might arise in any pathological condition accounted for 2.55%. Two miRNAs, hsa-miR-320a and hsa-miR-877-3p, in PEX glaucoma were involved in all GO categories associated with biological processes of autophagy, apoptosis, and neurogenesis. During the process of randomly selected GO category analysis, only hsa-miR-30d-5p, hsa-miR-320a, and hsa-miR-877-3p were included among significantly differentially expressed miRNAs in patients with PEX glaucoma. Many significantly differentially expressed miRNAs were not included in GO category analysis, indicating that these miRNAs were not previously reported in the field of miRNA.

In NTG, cell death-related categories including autophagy (4.63%) and apoptosis (2.80%) also occupied the greatest proportion (7.43%) (Fig. 4b). Categories associated with neurogenesis (5.88%), inflammatory response (3.91%), and aging (3.21%) also presented significant proportions. Categories associated with cellular functions such as differentiation, migration, and proliferation that might arise in any pathological condition accounted for 6.85%. Three miRNAs (hsa-let-7c-5p, hsa-miR-192-5p, and hsa-miR-375) in NTG were involved in all GO categories related to biological processes of autophagy, apoptosis, and neurogenesis. During the process of randomly selected GO category analysis, most of those significantly differentially expressed miRNAs were included except for hsa-miR-4639-5p and hsa-miR-6777-5p in NTG.

**Figure 5:**
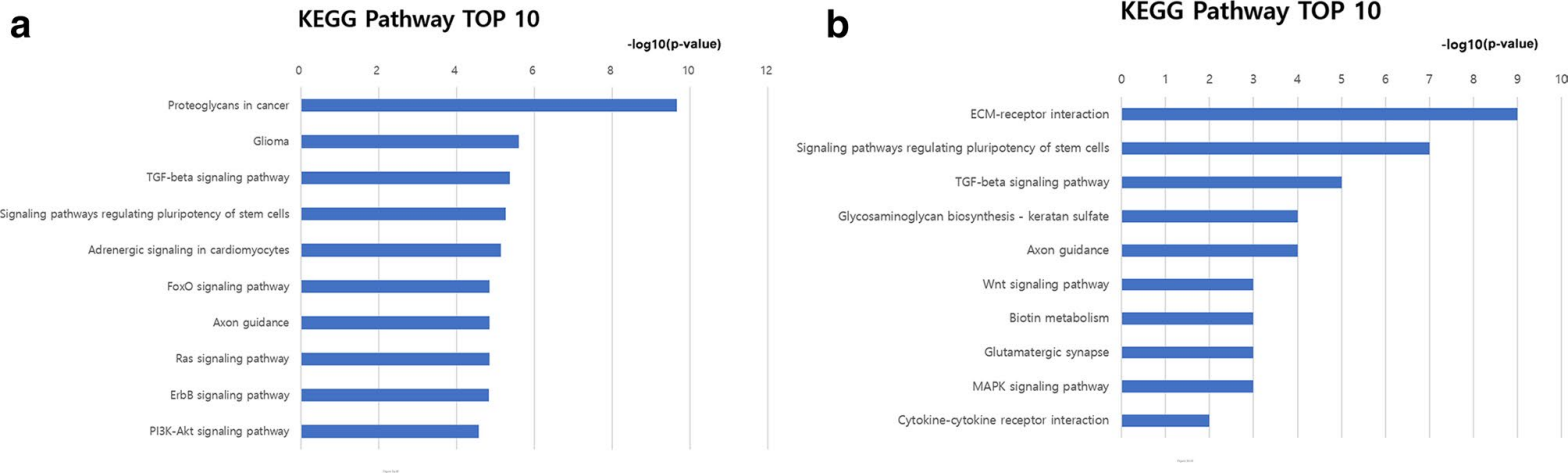
KEGG pathway. Enrichment score was represented as − log10 (*P* value). The higher the enrichment score value, the more significant the pathway. (**a**) Pathways related to proteoglycans in cancer (9.66, enrichment score, − log10 (*P* value)), glioma (5.59), TGF-beta signaling pathway (5.37) and signaling pathways regulating pluripotency of stem cells (5.26) were significantly associated with significantly differentially expressed miRNAs in the AH of PEX glaucoma patients. Among them, proteoglycans in cancer showed the most significantly related KEGG pathway in PEX glaucoma. (**b**) Pathways related to ECM-receptor interaction (8.57, enrichment score, − log10 (*P* value)), signaling pathways regulating pluripotency of stem cells (6.56), and TGF-beta signaling pathway (5.10) were significantly associated with significantly differentially expressed miRNAs in the AH of NTG patients. Among them, ECM-receptor interaction showed the most significantly related KEGG pathway in NTG. Data were analyzed with DianaTools. KEGG: Kyoto Encyclopedia Genes and Genomes; PEX: pseudoexfoliation; ECM: extracellular matrix; NTG: normal tension glaucoma.

The leading Kyoto encyclopedia of genes and genomes (KEGG) pathways, including predicted gene targets of each miRNA, for PEX glaucoma are presented in Table 3. The analysis of gene-annotation enrichment was performed with the database for annotation, visualization, and integrated discovery (DAVID). Pathways related to proteoglycans in cancer (9.66, enrichment score, − log10 (*P* value)), glioma (5.59), TGF-beta signaling pathway (5.37), and signaling pathways regulating pluripotency of stem cells (5.26) were significantly associated with significantly differentially expressed miRNAs in the AH of PEX glaucoma patients. Among them, proteoglycans in cancer showed the most significantly related KEGG pathway in PEX glaucoma (Fig. 5a). Pathways such as adrenergic signaling in cardiomyocytes (5.14) and FoxO signaling pathway (4.84) were also associated with significantly expressed miRNAs.

**Table 3:** Significant KEGG Pathways Potentially Influenced by MicroRNAs in the Aqueous Humor of Pseudoexfoliation Glaucoma Patients.

KEGG pathway	*P* value (− log10)	Genes predicted as target	Related miRNAs
Proteoglycans in cancer	9.66	88	10
Glioma	5.59	34	9
TGF-beta signaling pathway	5.37	41	9
Signaling pathways regulating pluripotency of stem cells	5.26	66	11
Adrenergic signaling in cardiomyocytes	5.14	62	10
FoxO signaling pathway	4.84	64	9
Axon guidance	4.84	57	10
Ras signaling pathway	4.84	92	11
ErbB signaling pathway	4.83	44	10
PI3K-Akt signaling pathway	4.57	135	11

KEGG: Kyoto encyclopedia of genes and genomes, NTG: normal-tension glaucoma, ECM: extracellular matrix.

KEGG pathways for NTG are presented in Table 4. Pathways related to ECM-receptor interaction (8.57, enrichment score, − log10 (*P* value)), signaling pathways regulating pluripotency of stem cells (6.56), and TGF-beta signaling pathway (5.10) were significantly associated with significantly differentially expressed miRNAs in the AH of NTG patients. Among them, ECM-receptor interaction showed the most significantly related KEGG pathway in NTG (Fig. 5b). Pathways such as glycosaminoglycan biosynthesis—keratan sulfate (3.81), and axon guidance (3.81) were also associated with significantly expressed miRNAs.

**Table 4:** Significant KEGG pathways potentially influenced by MicroRNAs in the Aqueous Humor of Normal Tension Glaucoma Patients.

KEGG pathway	*P* value (− log10)	Genes predicted as target	Related miRNAs
ECM-receptor interaction	8.57	21	9
Signaling pathways regulating pluripotency of stem cells	6.56	49	11
TGF-beta signaling pathway	5.10	25	10
Glycosaminoglycan biosynthesis—keratan sulfate	3.81	6	7
Axon guidance	3.81	36	10
Wnt signaling pathway	3.22	40	11
Biotin metabolism	2.94	1	1
Glutamatergic synapse	2.94	27	12
MAPK signaling pathway	2.93	63	11
Cytokine-cytokine receptor interaction	2.32	39	10

KEGG: Kyoto encyclopedia of genes and genomes, NTG: normal-tension glaucoma, ECM: extracellular matrix.

## Discussion

To the best of our knowledge, the present study was the first to report miRNAs significantly differentially expressed in individual AH samples between PEX glaucoma and NTG patients compared to controls in a single ethnic group of Koreans. The current study did not pool AH samples in spite of the scanty volume of each sample. We performed RNA sequencing to identify microRNAs in each AH sample. A total of 334 miRNAs in all PEX glaucoma AH samples and 291 miRNAs in all NTG AH samples were detected by RNA sequencing. We found two significantly upregulated miRNAs (hsa-miR-30d-5p, hsa-miR-320a) and ten significantly downregulated miRNAs (hsa-miR-3156-5p, hsa-miR-4458, hsa-miR-6717-5p, hsa-miR-6728-5p, hsa-miR-6834-5p, hsa-miR-6864-5p, hsa-miR-6879-5p, hsa-miR-877-3p, hsa-miR-548e-3p, and hsa-miR-6777-5p) in PEX glaucoma patients compared to controls. In NTG patients, we found ten significantly upregulated miRNAs (hsa-let-7a-5p, hsa-let-7c-5p, hsa-let-7f.-5p, hsa-miR-192-5p, hsa-miR-10a-5p, hsa-miR-10b-5p, hsa-miR-375, hsa-miR-4510, hsa-let-7b-3p, and hsa-miR-222-3p) and two significantly downregulated miRNAs (hsa-miR-4639-5p, and hsa-miR-6777-5p) compared to controls. Only hsa-miR-6777-5p, which was significantly down-regulated, was found in common between PEX glaucoma and NTG. Most of other individual miRNAs significantly differentially expressed compared to controls were different between PEX glaucoma and NTG. Moreover, some of those significantly differentially expressed miRNAs were regulated in an opposite way of up/down-regulation between PEX glaucoma and NTG groups compared to controls.

It is known that miRNAs play a significant role in the post-transcriptional modulation of gene expression. They are also involved in cellular functions such as cellular growth, differentiation, and cell death^27^. The human genome has about ~ 2500 mature miRNAs that modulate the expression of more than 60% of all protein-coding genes^13,28^.

In a previous study, Drewry et al.^21^ have reported differentially expressed miRNAs in the AH of patients, mainly Caucasians, with PEX glaucoma or POAG. They found that five miRNAs (miR-122-5p, miR-3144-3p, miR-320a, miR-320e and miR-630) were significantly differentially expressed between PEX glaucoma and controls^21^. In the present study, all subjects were Koreans. Thus, significantly differentially expressed miRNAs between PEX glaucoma and controls were different from those of the previous study. However, one miRNA, hsa-miR-320a, was in common with the previous study. This is interesting because we can speculate that hsa-miR-320a may contribute to the pathogenesis of PEX glaucoma irrespective of all ethnicities. Additionally, it may also indicate the validity of our results in PEX glaucoma. However, in the previous study, hsa-miR-320a was significantly down-regulated in PEX glaucoma compared to control, while it was significantly up-regulated in our study. Although these results are contradictory, we can still assume that hsa-miR-320a could play a role in PEX glaucoma in both Caucasians and Koreans.

There are several reports regarding hsa-miR-320a from blood samples of patients with different diseases. However, hsa-miR-320a has not been implicated in glaucoma yet. In an international multicenter study by Regev et al., serum hsa-miR-320a was significantly up-regulated in multiple sclerosis (MS) patients compared to healthy controls^29^. MS is an autoimmune disorder that injures the central nervous system^30,31^. It has been reported that increased expression of hsa-miR-320a, hsa-miR-320b, and hsa-miR-320c might be related to MS pathophysiology, with overexpression of miR-320 found in MS lesions^32^. Many previously reported hsa-miR-320 targets could be involved in MS progression and other diseases, for instance, cancer. These targets contain CD71, MCL-1, MMP-9, NRP1, HDAC4, B-catenin, and MAPK^33–40^. miR-320 is also significantly increased in the sera of patients with Alzheimer disease and asthma in comparison with healthy control samples (unpublished data^29^ and Raheja et al.^41^). Based on these findings, up-regulation of hsa-miR-320a might be involved in neurologic diseases since MS, Alzheimer disease, and glaucoma are all neurologic disorders. Moreover, one of major GO category in PEX glaucoma was neurogenesis (2.35%) and one of the most significant related KEGG pathways in PEX glaucoma was glioma in our study. In this regard, it may partly explain results of our study in PEX glaucoma.

Drewry et al. have also reported three significantly different miRNAs, miR-125b-5p, miR-302d-3p, and miR-451a, between POAG and controls from the AH^21^. These significantly differentially expressed miRNAs of POAG in Caucasians are also different from our study results. They did not overlap with those of NTG in Asians (Koreans), which have not been reported before.

A recent study has reported microRNAs in the AH from different types of glaucoma patients in Poland by real-time polymerase chain reaction method^42^. They found that hsa-miR-6722-3p and hsa-miR-184 were more frequently expressed in PEX glaucoma and hsa-miR-1260b was more frequently expressed in POAG^42^. These miRNAs in PEX glaucoma in Polish are different from our significantly differentially expressed miRNAs in PEX glaucoma compared to controls in Koreans by RNA sequencing. Moreover, hsa-miR-1260b in POAG in Polish is also different from those significantly differentially expressed miRNAs in NTG compared to controls in Koreans in our study. Based on results of these several studies, significantly differentially expressed miRNAs might be different between different types of glaucoma. Moreover, these differentially expressed miRNAs may also differ between different ethnicities.

One common miRNA, hsa-miR-6777-5p, was significantly differentially down-regulated in both PEX glaucoma and NTG. This suggests that this miRNA might be related to the pathogenesis of both PEX glaucoma and NTG in Asians (Koreans). However, there is no previous report on this miRNA. Since PEX glaucoma and NTG are both glaucoma, there could be a common miRNA that is involved in the pathogenesis of glaucoma. However, in our study, only one significant miRNA was in common between PEX glaucoma and NTG. Most of other individual significant miRNAs showed different expression patterns between the two. Moreover, five significantly differentially expressed miRNAs in PEX glaucoma compared to control showed opposite up/down regulated expression between PEX glaucoma and NTG. It was found that the same miRNA, hsa-miR-320a, was significantly up-regulated in PEX glaucoma while it was significantly down-regulated in NTG compared to controls (Fig. 3a). There were also two significantly differentially expressed miRNAs (hsa-miR-10a-5p, and hsa-miR-4639-5p) in NTG compared to control which showed opposite up/down regulated expression between NTG and PEX glaucoma (Fig. 3b). We may assume that the action of related miRNAs and the pathogenesis may differ between different types of glaucoma. It may further suggest that the pathogenic mechanism is different between primary glaucoma and secondary glaucoma. Furthermore, it indicates that future gene therapy should target different miRNAs according to different types of glaucoma.

Not only individual miRNAs were different between PEX glaucoma and NTG, but also individual related KEGG pathways were different between them. The most significantly related KEGG pathways in PEX glaucoma was proteoglycans in cancer (*P* value (− log10) = 9.66), followed by glioma (*P* value (− log10) = 5.59) and TGF-beta signaling pathway (*P* value (− log10) = 5.37). While in NTG, the most significantly related KEGG pathways was ECM-receptor interaction (*P* value (− log10) = 8.57), followed by signaling pathways regulating pluripotency of stem cells (*P* value (− log10) = 6.56) and TGF-beta signaling pathway (*P* value (− log10) = 5.10). Other than the top KEGG pathway in PEX glaucoma, which was proteoglycans in cancer, other pathways showed similar *P* values (− log10) (Fig. 5a). This suggests that the top KEGG pathway may contribute to the pathogenesis of PEX glaucoma more significantly than other pathways of similar degrees. In NTG, other than the top three KEGG pathways, other pathways showed much smaller or similar *P* values (− log10), (Fig. 5b). Thus, the top 3 KEGG pathways seem to contribute to the pathogenesis of NTG more significantly than others. In these regards, major related pathway and pathogenesis may differ between different types of glaucoma or between secondary glaucoma and primary glaucoma.

Proteoglycans are proteins that are heavily glycosylated. They are major components of animal ECM^43^. PEX material is highly cross-linked glycoprotein–proteoglycan aggregate comprised of a various proteins, such as fibronectin, laminin, vitronectin, fibrillin-1, extracellular chaperone clusterin, latent transforming growth factor-b (TGF-b) binding protein (LTBP), and cross-linking enzymes like lysyl oxidase-like 1 (LOXL1)^44–46^. Most of these proteins are present in ECM of normal eyes. These aggregates are synthesized intracellularly in many different types of cells in the anterior segment. These materials are then released into the extracellular space and deposited around the cells that produce these materials^4^. The accumulation of PEX material in the ECM of tissues can result in alteration of metabolism, thus, disturbing the structure and function. Considering that PEX syndrome is a disorder of the ECM and that PEX material is associated with proteoglycans, it seemed reasonable that the most significantly related top KEGG pathway in PEX glaucoma was “proteoglycans” in cancer in the present study.

Malignant cancer cells can breach away from the primary tumor, attach to, and degrade proteins that comprise the surrounding ECM, which separates the tumor from adjacent tissues. Through degrading these ECM proteins, cancer cells can break the ECM and escape^47^. The involvement of ECM during the process of metastasis in cancer might have led to the result that the most significant KEGG pathway related with PEX glaucoma was “proteoglycans in cancer” in the present study.

Transforming growth factor-beta (TGF-b) signaling pathway was one of top 3 related KEGG pathways in both PEX glaucoma and NTG in our study. Many previous studies have suggested that TGF-b plays a major role in the pathogenesis of glaucoma^48–52^. Numerous studies have revealed increased level of TGF-b in the AH of POAG patients^48–52^. TGF-b signaling has been invloved in the pathophysiology of vascular, neurodegenerative, and ocular diseases, along with remodeling of ECM^53,54^. The overlap in the pathogenesis of glaucoma and cellular and tissue reactions due to TGF-b suggests that disturbed TGF-b signaling could be related with the pathogenesis of glaucoma. Moreover, TGF-b1 and TGF-b2 can increase ECM production and inhibit the degradation of ECM that is present^55^. The imbalance of matrix degrading enzymes aroused by elevated TGF-b might contribute to the accumulation of PEX material in PEX syndrome, eventually bringing about the development of PEX glaucoma^56,57^. Moreover, since PEX material is glycoprotein–proteoglycan aggregate composed of various proteins including latent TGF-b binding protein (LTBP)^44–46^, TGF-b is considered to be closely related to PEX glaucoma. These reports could partly explain results of our study that TGF-b signaling pathway was one of top three significant related pathways, especially in PEX glaucoma associated with the accumulation of PEX material in ECM.

Individual biologic processes in gene ontology category were different between PEX glaucoma and NTG. However, cell death related mechanisms such as apoptosis, autophagy, and neurogenesis, inflammatory response comprised a major proportion in both PEX glaucoma and NTG. These biologic processes might be commonly associated with the pathogenesis of both types of glaucoma. However, autophagy dysfunction has been suggested in relation to the pathogenesis of PEX syndrome at cellular organelles level^12^. Autophagy is accountable for clearance of protein aggregates, which is vital to cellular homeostasis^58^. Considering the significance of autophagic clearance of protein aggregates, autophagy-related gene variants might be associated with the pathogenesis of PEX syndrome^59^. It has been reported that Tenon’s cells from PEX glaucoma patients compared to POAG counterparts have different phenotypic signs of decreased clearance of autophagosomes attributable to autophagy dysfunction^60^. Other than apoptosis, as a common pathological feature of RGC death^3^ in all glaucoma, autophagy might be more important in PEX glaucoma. However, the percentage of GO category from PEX glaucoma in Fig. 4a was not very high and the number of miRNAs was just a few. This was because only three significantly differentially expressed miRNAs were included in the GO analysis while most (nine) miRNAs were not included. It implied that these miRNAs were not previously reported in the field of miRNA. It further indicates that those miRNAs found in this study can be rather new in this field, especially related to PEX glaucoma.

Individual biological processes or KEGG pathways associated with PEX glaucoma or NTG need to be confirmed thorough further clinical and experimental studies. However, our study was unique in that significantly differentially expressed microRNAs were demonstrated in the individual sample of AH between PEX glaucoma and NTG patients compared to controls in a single ethnic group of Koreans. The underlying miRNA-associated pathways may further suggest novel targets for the pathogenesis of PEX glaucoma or NTG in Koreans.

Aging was one of the GO biological process categories possibly affected by microRNAs in the AH of NTG patients, as well (Fig. 4a, b). Since glaucoma is an age-related disease and the prevalence of glaucoma increases with age as shown in numerous population-based studies in the world and also in Asians including Koreans^22^, results of the current study seem reasonable.

Other biologic processes categories of GO influenced by microRNA in the AH of NTG patients compared to control included cellular cycle, migration, proliferation, differentiation, secretion, DNA repair, and angiogenesis. These categories might function in common with any pathological conditions. They may not happen only in glaucoma. In this aspect, such categories were not described in detail in the current study.

This exploratory study was limited by the relatively small sample size and the small volume of AH samples. However, it emphasized the potential for further research in this field. As the volume of the AH samples was not enough for all samples to undertake qPCR for validation, only hsa-let-7c-5p underwent qPCR. Results of validation qPCR were consistent with results of RNA sequencing for both PEX glaucoma and NTG patients. The expression of hsa-let-7c-5p was only significantly up-regulated in NTG patients (33.03 ± 2.84-fold), but not in PEX glaucoma patients (0.90 ± 0.30-fold) compared to controls. These results partly imply the validity and the reliability of our RNA sequencing results. The impact of hypotensive topical medications on microRNA expression within the AH of glaucoma patients has not been reported yet. The influence of using diverse hypotensive anti-glaucoma topical medications on our results is not known. Further studies with large numbers of samples would be advantageous in controlling the use of topical medications. However, glaucoma patients usually take hypotensive topical medications, especially when they are decided to undergo ophthalmic surgery unless they are found naïve in clinic. Under circumstances that it is not ethical to just obtain AH of patients that are not undergoing ophthalmic surgery in operation rooms only for research nor to stop medications for glaucoma patients just for research, it would not be easy to exclude the impact of hypotensive topical medications in accordance with the approval of IRB. NTG patients were medically well controlled and AH was obtained during cataract surgery, whereas most of PEX glaucoma patients underwent trabeculectomy due to uncontrolled IOP despite full medication. Therefore, baseline MD was much worse in the PEX glaucoma group (− 18.59 ± 10.54 dB) than in the NTG group (− 5.85 ± 4.81 dB). This difference in the stage of glaucoma might have influenced results of the current study. However, PEX glaucoma usually presents with aggressive form with medically poorly controlled high IOP frequently requiring filtering surgery such as trabeculectomy. Characteristics of PEX glaucoma are considered to be reflected in the demographics of our study.

In conclusion, we found significantly differentially expressed microRNAs in the AH between PEX glaucoma and NTG patients compared to controls in a single ethnic group of Asians (Koreans), which has not been previously reported. Differentially expressed miRNAs between PEX glaucoma and NTG samples compared to controls indicate possible roles of miRNA in the pathogenesis of glaucoma. Differentially expressed miRNAs between PEX glaucoma and NTG patients suggest that pathogenic mechanism and the individual role of miRNA might differ between different types of glaucoma or between secondary glaucoma and primary glaucoma. Future studies with more case numbers are required to reach conclusive answers. Our results can be further studied on a larger scale to better investigate the Asian population. Moreover, microRNAs in individual AH might have potential as novel biomarkers and also new targets for the pathogenesis of PEX glaucoma and NTG.

## Methods

### Ethics statement

This study was carried out in accordance with the tenets of the Declaration of Helsinki for research regarding human subjects. The present study was approved by the Institutional Review Board of Gyeongsang National University Changwon Hospital, Gyeongsang National University, School of Medicine (GNUCH-2019-06-001-002). Written informed consent was acquired from all subjects included in the current study. All methods were performed according to relevant guidelines and regulations.

### Diagnosis of NTG and PEX glaucoma

Subjects were evaluated in the glaucoma clinic at Gyeongsang National University Changwon Hospital by a single glaucoma specialist (H–K. C.). NTG was defined as the following: an IOP of ≤ 21 mmHg without treatment and findings of glaucomatous optic disc injury and corresponding VF defects, an open angle inspected by gonioscopy, and no other cause of optic disc impairment than glaucoma.^61^ PEX glaucoma was defined with the presence of PEX material at the margin of the pupil and on the anterior lens capsule after maximal pupil dilatation and all of the following: an initial intraocular pressure of at least 22 mmHg, glaucomatous optic disc changes, visual field defects consistent with optic nerve damage, and no evidence of other conditions causing secondary glaucoma^7^. All subjects went through standard ophthalmic examinations including slit-lamp biomicroscopy, gonioscopy, and funduscopy.

### Patient selection and acquisition of aqueous humor samples

Samples of AH were acquired from patients who received uneventful phacoemulsification for elective cataract surgery or trabeculectomy after acquiring written informed consent. Eleven PEX glaucoma patients, age-matched eight NTG patients, and seven control subjects agreed to participate in the current study. About 80 to 120 µl of AH was acquired by anterior chamber paracentesis with a 30-gauge needle prior to the main cataract incision at the start of cataract surgery or during the process of paracentesis in trabeculectomy. Anterior chamber paracentesis was achieved under aseptic sterile conditions in the operating room. The AH was acquired without trauma to subjects, hence eliminating any probability of contamination with blood or cellular debris. All acquired samples were utterly anonymized, instantly snap-frozen with liquid nitrogen, and thereafter transported to research laboratories. Clinical data were collected from electronic medical records in an entirely anonymized way. Obtained clinical data were eye laterality, age, baseline IOP, sex, topical medications used, and other ocular comorbidities.

### RNA isolation

Total RNA was extracted using Trizol LS reagent (Invitrogen, Carlsbad, CA, USA) following the manufacturer’s instructions. The quality of RNA was assessed by an Agilent 2100 bioanalyzer using an RNA 6000 Pico Chip (Agilent Technologies, Amstelveen, The Netherlands). RNA quantification was performed using a NanoDrop 2000 Spectrophotometer system (Thermo Fisher Scientific, Waltham, MA, USA).

### Library preparation and RNA sequencing

Libraries were constructed employing NEBNext Multiplex Small RNA Library Prep kit (New England BioLabs, Inc., Ipswich, MA, USA) for control and test RNAs, in accordance with the manufacturer’s instructions^62^. Briefly, 180 pg of total RNA from each AH sample was employed to ligate with 1 µg of adaptors. Afterwards, cDNA was synthesized using reverse-transcriptase with adaptor-specific primers. PCR was done for library amplification. Libraries were cleaned-up using a QIAquick PCR Purification Kit (Qiagen, Inc, Germany) and AMPure XP beads (Beckman Coulter, Inc., Pasadena, CA, USA). The yield and size distribution of small RNA libraries were assessed with an Agilent 2100 Bioanalyzer instrument and High-sensitivity DNA Assay (Agilent Technologies, Inc., USA). High-throughput sequences were generated with a NextSeq500 system by single-end 75 sequencing (Illumina, San Diego, CA, USA).

### microRNA validation by quantitative real-time PCR

cDNA synthesis and real-time PCR were conducted with an miScript PCR system (Qiagen, Venlo, The Netherlands). cDNA was synthesized from 357 pg of RNA using the miScript II RT Kit with HiSpec buffer according to the manufacturer’s instructions. The miRNA cDNA was amplified with the following primer pair: hsa-let-7c-5p (Hs_let-7c_1, MS00003129) and internal control hsa-U6 (Hs_RNU6-2_11, MS00033740). Real-time PCR was performed on a StepOnePlus™ Real-Time PCR System (Applied Biosystems, Foster City, CA, USA) employing a QuantiTect SYBR Green PCR Master mix and an miScript Primer Assay (Qiagen) according to the manufacturer’s instructions. Thermal cycling conditions were: 95 °C for 15 min followed by 40 cycles of 94 °C for 15 s, 55 °C for 30 s, and 70 °C for 30 s. Data were analyzed with StepOne software v2.2.2 (Applied Biosystems). The level of expression of each miRNA was normalized to the median Ct value and calculated using the 2^−ΔΔCt^ method.

### Data analysis

Sequence reads were mapped using the bowtie2 software tool to obtain bam files (alignment file). Mature miRNA sequences were used as references. Read counts were employed to detect expression levels of miRNAs. Read counts mapped on mature miRNA sequences were extracted from the alignment file using bedtools (v2.25.0)^63^ and Bioconductor (EdgeR package) that employed R statistical programming language (R Development Core Team, 2011, version 3.2.2). For quality control, trimming was performed with a BBDuk tool. Illumina TruSeq adapter was employed and phred quality threshold were over 20. The quantile normalization method was used for the comparison between samples. For miRNA target study, DianaTools-mirPath v.3 (http://diana.imis.athena-innovation.gr/DianaTools/index.php?r=site/page&view=software) was used. DianaTools, miRTarBase (http://mirtarbase.mbc.nctu.edu.tw/php/search.php), miRWalk 2.0. (http://zmf.umm.uni-heidelberg.de/apps/zmf/mirwalk2/), and TargetScan (http://www.targetscan.org/vert_72/) were applied to predict miRNA targets. Relevant KEGG pathways were analyzed in compliant with previous studies from Kanehisa Laboratories^64–67^. Data were demonstrated with ExDEGA v1.2.1.0 software (EBIOGEN, Inc., Seoul, Korea).

### Statistical analysis

Data of microRNA validation are shown as mean ± standard error of the mean (S.E.M.). The analysis for the validation was performed with Unpaired Student’s t-test (Prism 5; GraphPad Software, La Jolla, CA, USA). *P* < 0.05 was regarded to demonstrate a statistically significant difference. Enrichment *P* values were modified for false discovery rate (FDR)^68^.
